# The affinity–efficacy problem: an essential part of pharmacology education

**DOI:** 10.1098/rsos.240487

**Published:** 2024-07-24

**Authors:** James P. Higham, David Colquhoun

**Affiliations:** ^1^ Department of Pharmacology, University of Cambridge, Tennis Court Road, Cambridge CB2 1PD, UK; ^2^ Neuroscience, Physiology and Pharmacology, University College London, London WC1E 6BT, UK

**Keywords:** agonism, ligand binding, receptor theory, affinity, efficacy

## Abstract

A fundamental mistake in receptor theory has led to an enduring misunderstanding of how to estimate the affinity and efficacy of an agonist. These properties are inextricably linked and cannot be easily separated in any case where the binding of a ligand induces a conformation change in its receptor. Consequently, binding curves and concentration–response relationships for receptor agonists have no straightforward interpretation. This problem—the affinity–efficacy problem—remains overlooked and misunderstood despite it being recognized in 1987. To avoid the further propagation of this misunderstanding, we propose in this review that the affinity–efficacy problem should be included in the core curricula for pharmacology undergraduates proposed by the British Pharmacological Society and the International Union of Basic and Clinical Pharmacology (IUPHAR).

## 1. Introduction

In 1956, R.P. Stephenson proposed a modification of receptor theory which has had a lasting influence [1]. He said that in addition to the affinity of a drug for a receptor, an extra parameter was needed to describe the action of an agonist. This extra parameter he called *efficacy*, which he defined as a measure of the agonist to produce a response once it became bound. This idea provided a unified understanding of agonists, partial agonists and antagonists. However, a mistake in his framing of the problem resulted in a fundamental misunderstanding of how to estimate values of affinity and efficacy, a misunderstanding that still pervades pharmacology today. The resolution of this mistake, which was uncovered in 1987 [2], demonstrated, somewhat counterintuitively, that agonist binding depends on both its affinity *and* its efficacy [3]; a consideration which remains all-too-often overlooked in the teaching of pharmacology and the pharmacological literature. This review is intended to explain the problem, commonly referred to as the affinity–efficacy, or binding-gating, problem.

## 2. A simple agonist mechanism

The problem in question is best illustrated by first considering the framework proposed by Stephenson [1]. He proposed that the fractional receptor occupancy (*p*) for an agonist (A) with a dissociation equilibrium constant, *K*_A_, is given by the Langmuir equation


(2.1) $$p=\frac{\left[A\right]}{\left[A\right]\;+\;K_{A}},$$

where [A] is the equilibrium concentration of the agonist and *K*_A_ is defined as the ratio of the rate constants for dissociation, *k*_off_, and association, *k*_on_, of the ligand. This dissociation equilibrium constant, *K*_A_, has units of concentration, so the smaller the value, the greater the affinity. We shall often refer to the affinity as measured by *K*_A_. Strictly speaking, we should refer to the affinity as measured by 1/*K*_A_, but concentrations are much easier to understand than reciprocal concentrations, so the former wording will be used throughout.

Stephenson postulated that the response to the agonist could be written as a function of the product of the occupancy and the efficacy of the agonist. The function was unknown but assumed to be the same for all agonists. Efficacy was an empirical number between zero and infinity; no physical interpretation of efficacy was proposed. Crucially, Stephenson assumed that affinity and efficacy could be separated experimentally using equilibrium measurements of agonist binding and agonist-evoked responses, an assumption that proved to be fatal for his approach.

The Langmuir equation, equation (2.1), which was first derived by Hill in 1909 [4], describes the binding of a ligand to identical, independent binding sites at equilibrium. That would be sufficient to describe the binding of a competitive antagonist to a receptor, given that the antagonist does not produce a conformational change in the receptor, i.e. only the vacant and bound inactive states of the receptor are present [5]. However, it cannot be expected to describe the action of an agonist, which must produce a conformational change in the receptor to produce a response. Neither can the Langmuir equation account for the phenomenon of partial agonism. The maximum occupancy at high ligand concentration is always one, but not all agonists elicit the same maximum response when all receptors are occupied.

The simplest possible mechanism that overcomes these problems was proposed a year after Stephenson’s paper, by del Castillo & Katz [6]. Stephenson had hoped to solve the problem without postulating a mechanism for agonist action, whereas del Castillo & Katz proposed a simple mechanism. They added an extra state: after the agonist had bound to the receptor, it could isomerize to an active conformation. If we denote the agonist molecule as A, and the receptor as R (in its inactive conformation) or R* (in its active conformation), the del Castillo–Katz mechanism can be written thus


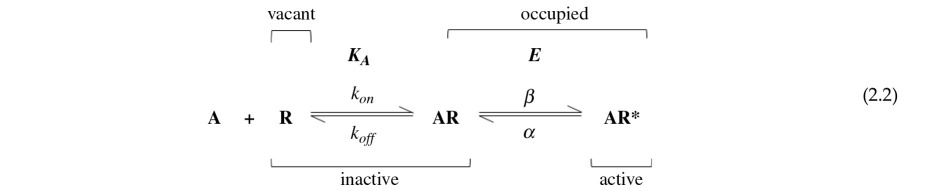


The equilibrium constant for the binding reaction is *K*_A_, as before. The equilibrium constant for the isomerization between the inactive and active conformations is denoted *E* because this is a measure of the agonist’s efficacy. *E* is defined as the ratio of the rate constants for isomerization to (*β*) and from (*α*) the active state, AR*.

The mechanism in equation (2.2) is directly analogous to the Michaelis–Menten mechanism [7], which was published in 1913 and has been used as a simple description of the rate of action of enzymes ever since. In fact, the mechanism in equation (2.2) is even simpler than the Michaelis–Menten case, in which the last step of the reaction is usually assumed to be irreversible, so it cannot attain true equilibrium, but only a quasi-steady state.

In equation (2.2), both of the states AR and AR* are bound by agonist, so to calculate the receptor occupancy that would be found in an equilibrium agonist binding experiment, it is necessary to add the occupancies of these two states, *p*_AR_ and *p*_AR*_. Application of the law of mass action to equation (2.2) shows that


$$p_{AR}=\frac{\left[A\right]p_{R}}{K_{A}},$$

$$p_{AR^{*}}=Ep_{AR},$$

where *p*_R_ is the fractional occupancy of the vacant state. Consequently, the total fraction of receptors bound by agonist (*p*_bound_) is


(2.3) $$p_{bound}=p_{AR}+p_{AR^{*}}=\frac{\left[A\right]}{\left[A\right]\;+\;K_{eff}}.$$

The derivation of this result is included in the electronic supplementary material and is explained in detail in two videos [8,9]. The result looks exactly like the Langmuir equation, (2.1), except it contains a different equilibrium constant—not the affinity, $K_{A}$ , but rather an *effective* equilibrium constant, $K_{eff}$ , defined as


(2.4) $$K_{eff}\equiv \frac{K_{A}}{1\;+\;E}.$$

The equilibrium constants for a single step in the reaction, like $K_{A}$ , are called microscopic constants, whereas constants that describe the net result of more than one step, like $K_{eff}$ , are called macroscopic constants. The word affinity is, therefore, ambiguous. It may refer to *K*_A_, the equilibrium constant for the binding step in equation (2 .2 ) or *K*_eff_, defined in equation (2.4), which is the equilibrium constant measured in an agonist binding experiment, i.e. the concentration of agonist required for half-maximal binding. Only the former, *K*_A_, gives information about agonist binding to its binding site. The latter, *K*_eff_, depends also on *E*, the equilibrium constant for the isomerization between the inactive and active conformations, which can be affected by a change anywhere in the receptor structure. Hereinafter, *K*_A_ and *K*_eff_ will be referred to as microscopic and macroscopic affinities, respectively. For the purposes of understanding the relationship between receptor structure and function, it is the former that is needed, but only the latter can be inferred from experimental observations (except for single ion channel experiments).

This shows that the binding of an agonist to a receptor, as measured by a ligand binding experiment, is dependent not only on its microscopic affinity but also on its efficacy. In Stephenson’s framework, microscopic affinity and efficacy are not (easily) separable as he had assumed. This conclusion also follows, more generally, from thermodynamic considerations: the principle of reciprocity states that if binding affects the equilibrium between R and R*, which is the case for an agonist, then the reverse must also be true (e.g. [10,11]).

### 2.1. Stephenson’s mistake

Stephenson postulated that the proportion of receptors that are occupied by an agonist at equilibrium depended only on *K*_A_ as in equation (2.1). In the 1980s, one of us (D.C.) asked Stephenson whether *occupancy* in this statement referred to that which would be measured in a ligand binding experiment. When Stephenson answered ‘yes’, it became obvious that he had made a fundamental error. As shown in equation (2.3), agonist binding depends on two different equilibrium constants; it depends on *both* its microscopic affinity *and* efficacy. The agonist binding curve is predicted to have the same shape as a Langmuir isotherm, but the concentration for half-maximal binding, $K_{eff},$ depends on both the affinity for the initial binding step, $K_{A}$ , and on efficacy, *E*, as shown in equation (2.4).

It is clear from equation (2.3) that the maximum occupancy is one. In other words, (virtually) all receptors become bound at a very high concentration of agonist. In the del Castillo–Katz mechanism, the response to the agonist is represented by the fraction of receptors in the active state, $p_{{AR}^{*}}$ . The sum of the fractional occupancies of each state in equation (2.2) must equal one, so the law of mass action implies that


(2.5) $$p_{AR^{*}}=\frac{Ec_{A}}{1+c_{A}+Ec_{A}},$$

where we have expressed the concentration of the agonist as a multiple of its equilibrium dissociation constant, by defining the dimensionless variable


(2.6) $$c_{A}\equiv \frac{\left[A\right]}{K_{A}}.$$

Equation (2.5) shows that the response to agonist for the del Castillo-Katz mechanism is the product of the agonist’s efficacy, *E*, and the occupancy of the bound but inactive, AR, state (see electronic supplementary material, e.g. equations S6, S12 and S13). Although similar to Stephenson’s postulate, this demonstrates the key problems with his approach: (i) it is not possible to find the occupancy of *only* the AR state in an agonist binding experiment, and (ii) the fraction of receptors in each state (and the total fraction of receptors occupied by agonist, equation (2.3)) depends on both *K*_A_ and *E* (see electronic supplementary material). Therefore, microscopic affinity and efficacy remain inextricably linked. At a high concentration of agonist, when all receptors are occupied, the maximum fraction of receptors in the active state, $p_{{AR}^{*}}^{max}$ , is given by the limit of equation (2.5) as the free agonist concentration becomes very high. In this case, all receptors are occupied (either as AR or as AR*), so $p_{{AR}^{*}}^{max}$ depends only on efficacy and is given by


(2.7) $$p_{AR^{*}}^{max}=\frac{E}{1+E}.$$

If the response is expressed relative to the maximum response, then it follows from equations (2.5), (2.6) and (2.7) that


(2.8) $$\frac{p_{AR^{*}}}{p_{AR^{*}}^{max}\;\;\;}=\frac{\left[A\right]}{\left[A\right]\;+\;K_{eff}}.$$

This is exactly the same as the ligand binding curve in equation (2.3), and so the response curve also gives information only about the macroscopic equilibrium constant, $K_{eff}$ .

**Figure 1.**
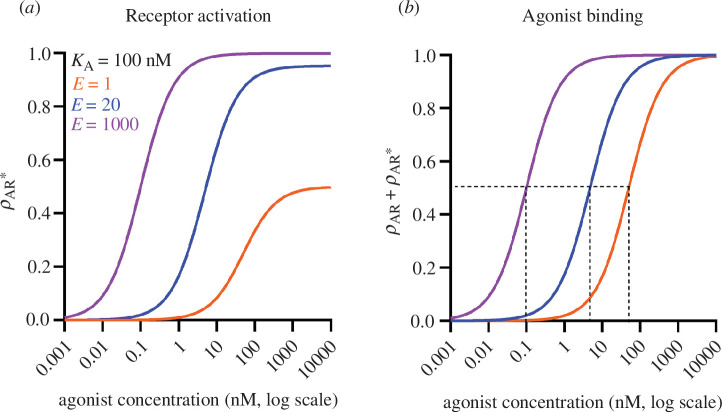
Illustration of the effect of changing efficacy for agonists with the same affinity. (*a*) The fraction of receptors occupying the active, AR*, state, calculated using equation (2.5), for agonists which all have the same microscopic affinity (*K*_A_ = 100 nM for all curves) but with differing efficacy (*E* = 1, orange curve; *E* = 20, blue curve; *E* = 1000, purple curve). (*b*) Binding of an agonist, calculated from del Castillo–Katz mechanism, equation (2.3). Left curve (purple), agonist with *K*_A_ = 100 nM and *E* = 1000. Middle curve (blue), agonist with the same microscopic affinity, *K*_A_ = 100 nM, but reduced efficacy, *E* = 20. Right curve (orange), agonist with the same microscopic affinity, *K*_A_ = 100 nM, but further reduced efficacy, *E* = 1. The grey dashed lines mark the concentrations at which half of the receptors are bound by agonist. These are *K*_eff_ = 100/(1000 + 1) ≈ 0.1 nM (purple curve), 100/(20 + 1) ≈ 4.8 nM (blue curve) and 100/(1 + 1) = 50 nM (orange curve). The reduction in efficacy has reduced binding by a factor of 4.8/0.1 = 48 fold and 50/0.1 = 500 fold, respectively, with no change in microscopic affinity.

For an agonist with *E* = 1, equation (2.7) shows that the maximum response evoked by a saturating concentration of agonist will correspond to half of the receptors being in the active state (AR*, figure 1*a*), e.g. half of the ion channels are open. The other half will be in the inactive but bound state (AR). In this case, the agonist will be obviously partial when compared with an agonist which evokes a larger maximum response. If *E* = 20, then the maximum response will correspond to 20/21 ≈ 95.2% of receptors in the active state. In most sorts of experiment, this would be indistinguishable from an agonist with *E* = 1000, which gives $p_{{AR}^{*}}^{max}$ = 1000/1001 ≈ 99.9% of receptors in the active state (figure 1*a*). So, it is impossible to distinguish between agonists with an efficacy greater than 10 or so by measuring the maximum responses they elicit. Beyond this point, increases in efficacy merely shift the log concentration–response curve to the left, an effect that is indistinguishable from an increase in microscopic affinity [1].

As Stephenson pointed out, an agonist with *E* = 1 will be obviously partial only if the response being measured is the fraction of receptors in the active state, as in the case for ion channels. For responses that are limited by things other than receptor saturation, it is possible that a maximum response will be elicited by activation of a small fraction of receptors. In this case, there are said to be *spare receptors*. For example, the maximum depolarization of the muscle endplate is achieved when only a small proportion of channels is open [12,13].

It might be thought that efficacy could be measured separately by observing the maximum response evoked by the agonist. Equation (2.7) could then give a value for *E*, and that could be used with an estimate of *K*_eff_ found from a ligand binding experiment to give an estimate of the (microscopic) affinity, *K*_A_, using equation (2.4). This method does indeed work in single ion channel experiments in which the maximum response can be measured on an absolute scale, though even in this case it works only for agonists with *E* less than 10 or 20—for any higher values of efficacy the maximum responses are sufficiently close to 100% as to be indistinguishable. However, in general, it cannot be used because of lack of knowledge of the relationship between the observed response and fraction of receptors in the active state and because maximum responses can be measured only relative to an arbitrary maximum response produced by a full agonist for which the efficacy, *E*, is unknown.

It is worth noting that for a ligand with *E* = 0, i.e. a competitive antagonist, then *K*_eff_ = *K*_A_; the binding of an antagonist will not affect the equilibrium between the R and R* conformations of the receptor. In this case, the Langmuir equation provides a good description of ligand binding, wherein only the vacant and bound inactive states of the receptor are present. That is why the Schild method [5] for the estimation of the affinity of a competitive antagonist works even when the relation between the number of active receptors and the observed response is not known. Stephenson tried to apply similar null methods to agonists, but this proved to be impossible.

### 2.2. Implications for structure–activity studies

All this has profound implications for the interpretation of structure–activity relationships for receptors. In equation (2.2), it is the equilibrium constant for binding, *K*_A_, which gives information about the agonist’s affinity for its binding site. The other equilibrium constant, *E*, tells you about the ability of the agonist to induce a conformation change in the receptor once it has become bound.

Both microscopic affinity and efficacy influence agonist binding, as shown in equation (2.3), and, crucially, *the ability to change conformation can be influenced by mutations in any part of the receptor protein*. That is why changes in agonist binding do not necessarily tell you anything about the binding site. The same is true for structure–activity relationships for agonists. If a change in the chemical structure of an agonist leads to a change in agonist binding, it is not possible to deduce, from the binding experiment alone, whether this is due to a change in agonist microscopic affinity or whether it results from a change in efficacy.

These ideas are illustrated in figure 1*b*, which shows the binding of an agonist, as calculated from equations (2.3) and (2.4). For each curve, the microscopic affinity is identical, *K*_A_ = 100 nM. The leftmost (purple) curve is for an agonist with very high efficacy, *E* = 1000. The middle curve (blue) is for an agonist with much reduced efficacy, *E* = 20 (this could be a different agonist on the same receptor or it could be the same agonist on a mutated receptor). The 48-fold reduction in macroscopic binding results entirely from reduced efficacy, so it does not necessarily tell us anything about the binding of the agonist to its binding site. The rightmost curve (orange) is for a low-efficacy agonist (*E* = 1), for which half-maximal binding is attained at a concentration of 50 nM. This represents a 500-fold reduction in observed (macroscopic) agonist binding, again with no change in *K*_A_.

Agonist binding experiments are, therefore, of limited use in the elucidation of microscopic agonist affinity, or of the location of the agonist binding site. One should be wary when reporting the results of agonist binding experiments as they provide only the effective equilibrium constant (macroscopic affinity), *K*_eff_, not the dissociation constant (microscopic affinity), *K*_A_. Concentration–response measurements provide very similar information (see electronic supplementary material).

All this being said, if the effects are big enough, it may be possible to justify conclusions about the binding site from such studies, but they need to be buttressed by quantitative arguments, as, for example, in Anson *et al*. [14]. Measurements of the change in microscopic affinity of competitive antagonists caused by mutations in the putative binding site (measured by the Schild method or in ligand binding experiments) provide stronger evidence for the location of the binding site, insofar as the antagonist does not cause a conformational change in the receptor [14].

## 3. A more complex agonist mechanism

**Figure 2.**
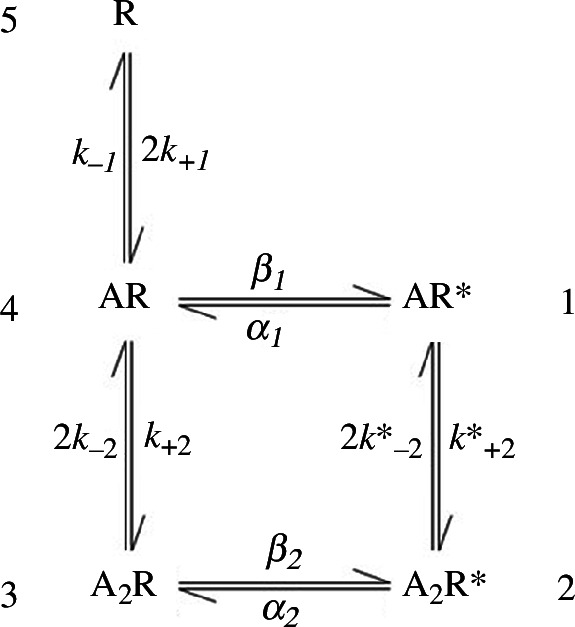
The mechanism for the nAChR proposed by Colquhoun & Sakmann [15]. Numbers adjacent to each state refer to their positions in the ***p*** and ***Q*** matrices (see text). Rate constants are given next to their respective transitions; some rate constants (e.g. the rate constant for the state 5 → 4, 3 → 4 and 2 → 1 transitions) are doubled because either one of two ligands may associate or dissociate during these transitions. This ensures that the rate constant refers to the binding site rather than the receptor as a whole.

The del Castillo–Katz mechanism is too simple to describe any real receptor, but it is sufficient to show the mistake in Stephenson’s framework. We will next consider a more realistic agonist mechanism, that for the interaction between acetylcholine (ACh) and related compounds and the muscle nicotinic acetylcholine receptor (nAChR) proposed in 1985 [15], as shown in figure 2. At the time, this was the best description of the action of ACh at the muscle nAChR, though it was later amended to include an intermediate inactive (shut) conformation which cast light on the mechanism of partial agonists [16].

The mechanism proposed in 1985 (figure 2) consists of three inactive states (channel is shut) and two active states (channel is open). While the binding of one agonist molecule can cause the opening of the channel, this reaction is quite unfavoured; the binding of two agonist molecules is far more likely to result in channel opening. To find the fractional occupancy of each state of the receptor, one could use the approach above for the del Castillo–Katz mechanism and apply the law of mass action to generate expressions for the occupancy of each state (see electronic supplementary material). While there is nothing wrong with this approach, it is somewhat cumbersome—and will become increasingly cumbersome for mechanisms with increasing numbers of states.

A more general way to solve for the fractional occupancies (for any mechanism) is to use matrix notation (section 3 in Colquhoun & Hawkes [17]). Matrices are, in fact, the only way to deal with this sort of problem in a general way. If you are unfamiliar with them, try this video: *Matrix algebra in 45 minutes* [18].

First, define a row vector containing the fractional occupancies, at time *t*, of each of the five states (***p*** (*t*)), numbered as in figure 2,


$$p\left(t\right)=\left[\begin{array}{ccccc} p_{1}\left(t\right) & p_{2}\left(t\right) & p_{3}\left(t\right) & p_{4}\left(t\right) & p_{5}\left(t\right) \end{array}\right].$$

Any reaction mechanism can be defined by its transition rate matrix, which is usually denoted as the ***Q*** matrix. This contains the rates for the transitions between each state. The off-diagonal element, $q_{ij}$ , in the *i*th row and *j*th column is the rate of the transition between states *i* and *j*. If there is no direct connection between two states, the transition rate between them is zero. The diagonal elements are defined such that the sum of each row in the ***Q*** matrix is zero (and, consequently, its determinant is also zero). For example, for the mechanism in figure 2, the ***Q*** matrix is:


$$Q=\left[\begin{array}{ccccc} -\left(\left[A\right]k_{+2}^{*}+\alpha_{1}\right) & \left[A\right]k_{+2}^{*} & 0 & \alpha_{1} & 0\\ 2k_{-2}^{*} & -\left(2k_{-2}^{*}+\alpha_{2}\right) & \alpha_{2} & 0 & 0\\ 0 & \beta_{2} & -\left(2k_{-2}+\beta_{2}\right) & 2k_{-2} & 0\\ \beta_{1} & 0 & \left[A\right]k_{+2} & -\left(\beta_{1}+\left[A\right]k_{+2}+k_{-1}\right) & k_{-1}\\ 0 & 0 & 0 & 2\left[A\right]k_{+1} & -2\left[A\right]k_{+1} \end{array}\right].$$

Using this notation, it is possible to rewrite the set of five simultaneous differential equations needed to describe the rate of change in the occupancy of each state (omitted here for brevity) simply as


$$\frac{dp\left(t\right)}{dt}=p\left(t\right)Q.$$

This equation is the same for any mechanism; all you need to do is to specify its ***Q*** matrix. At equilibrium (after infinite time), the occupancies do not change with time: d ***p*** (*t*)/d*t* = **0**, so


p(∞)Q=0,

where $p\left(\infty \right)$ is a row vector containing the fractional occupancies of each state at equilibrium. It is not immediately clear how to solve this for p(∞) because the ***Q*** matrix is singular (its determinant is zero) and so it cannot be inverted. Several ways exist to solve for $p\left(\infty \right)$ , but the most convenient for programming uses the augmented ***Q*** matrix [17]. This approach involves creating a new matrix (***S***) by augmenting the ***Q*** matrix with a unit column vector (***u***) which constrains the sum of the fractional occupancies to one. The ***S*** matrix can be written, in partitioned form, as


$$S=\left[\begin{array}{cc} Q & u \end{array}\right].$$

Hence, the transpose of ***S*** is


$$S^{T}=\left[\begin{array}{c} Q^{T}\\ u^{T} \end{array}\right].$$

Post-multiplying $p\left(\infty \right)$ by ***SS*** ^T^ gives


$$p\left(\infty \right)SS^{T}=p\left(\infty \right)QQ^{T}+p\left(\infty \right)uu^{T}.$$

As we are considering the system at equilibrium, $p\left(\infty \right)$ ***Q*** = 0, so the first term on the right-hand side is zero. And $p\left(\infty \right)$ ***u*** is the sum of the fractional occupancies of all states at equilibrium which must equal one, so the second term is $u^{T}$ . Therefore, this equation simplifies to


$$p\left(\infty \right)SS^{T}=u^{T}.$$

The determinant of ***SS*** ^T^ is non-zero, so it can be inverted, and post-multiplying both sides by (***SS*** ^T^)^-1^ gives the equilibrium fractional occupancies for each state as


(3.1) $$p\left(\infty \right)=u^{T}{\left(SS^{T}\right)}^{-1}.$$

Numerical examples of these calculations are given in the electronic supplementary material and in [9,19]. This approach enables one to find the equilibrium occupancies for all states of a receptor for *any* given mechanism.

Notice that the ***Q*** matrix contains all of the rate constants in the mechanism. This means that the binding of an agonist—as well as the response to an agonist (and the time constants for the approach to equilibrium)—depends on the *entire* reaction mechanism, including parameters describing the agonist’s efficacy, and not just the affinity for the initial binding step. Equation (3.1) is the general form of the ‘occupancy equation’: it gives the occupancies of every state at equilibrium, for any reaction mechanism, while equations (2.1) and (2.3) are special cases pertaining only to specific reaction mechanisms.

None of the receptor mechanisms discussed thus far have dealt with constitutive receptor activity or inverse agonism; a discussion of these phenomena and their relation to the affinity–efficacy problem is included in the electronic supplementary material.

## 4. The case of single ligand-gated ion channels

The ***Q*** matrix is at the heart of calculations of not only equilibrium properties, but also kinetics (the rate of approach to equilibrium), for both macroscopic and single molecule systems [17,19,20]. These can all be calculated from the ***Q*** matrix. It is possible to write a general equation that describes the rate at which the occupancy of each state approaches its equilibrium value


p(t)=p(0)exp(Qt),

where ***p*** (*t*) is a row vector containing the occupancies for each state at any time, *t*, and ***p*** (0) is a row vector containing the occupancies for each state at time *t* = 0. This simple-looking equation describes the kinetics for any mechanism (as long as ***Q*** is constant) but it is beyond the scope of this review—see [9,17,19,20].

In order to evaluate any of these equations, one needs numerical values for all of the rate constants in the reaction mechanism. The problem of estimating these values from experimental observations has, so far, been solved only for single ligand-gated ion channel recordings.

One problem in estimating the rate constants in a reaction mechanism resulted from the fact that very short channel openings or shuttings (shorter than approx. 20 µs) cannot be resolved in experimental recordings. This means that observed openings are too long (because of missed short shuttings) and observed shut times are extended by missed short openings. In 1990, an exact solution to this problem was found by Hawkes & Jalali [21–23]. This allowed the calculation of the probability distributions of the *observed* open and shut times, given values for the transition rates in the ***Q*** matrix. This, in turn, allowed the calculation of the probability (density) of observing the whole sequence of open and shut times in a recording, given putative values for the transition rates. This quantity is known as the likelihood of the set of putative transition rates. Their values are adjusted iteratively until the likelihood is maximized [24,25]. The maximum likelihood estimates of the transition rates, so found, can then be used to predict any other responses of the receptor and its ability to bind the ligand.

Simulations have shown that these methods can provide good estimates of the rate constants, and hence of the equilibrium constants, for mechanisms with up to 10 states and 14 free rate constants, under favourable conditions [26,27]. Thus, the problem of separating microscopic affinity and efficacy is solved.

**Figure 3.**
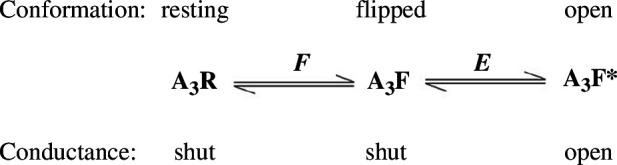
The case of a glycine receptor in the presence of a saturating concentration of agonist, so all three binding sites are fully occupied. The equilibrium constant for the isomerization between resting (A_3_R) and flipped (A_3_F) conformations is denoted as *F*, and the equilibrium constant for the isomerization between flipped and open (A_3_F*) conformations is denoted as *E*.

For example, methods like these have allowed us to postulate that there is a short-lived shut conformation (the ‘flipped’, or ‘primed’, conformation) that lies between the resting conformation and the open conformation of channels in the nicotinic receptor superfamily [16,27,28]. This implies that three states exist in the presence of a saturating agonist concentration, as shown in figure 3.

In this case, the maximum response corresponds to the maximum fraction of receptors in the open A_3_F* state, $p_{open}^{max}$ . This is determined by both of the equilibrium constants in figure 3. It can be written as


$$p_{open}^{max}=\frac{EF}{1+F+EF}=\frac{E_{eff}}{1+E_{eff}},$$

where we have defined an effective efficacy as


$$E_{eff}\equiv E\frac{F}{1+F}.$$

For a single receptor channel, the response can, under favourable conditions, be measured directly from experimental recordings as the fraction of time spent in open state. This is the only case in which the response to an agonist can be measured on an absolute scale rather than being measured relative to an arbitrary maximum.

Ever since 1957 [6], it had been assumed that partial agonists on ion channels were partial because the opening reaction was inefficient (*E* is small). But it was proposed by Lape *et al*. [16], that partial agonists are partial because the first step is inefficient (*F* is small), but the second step, the shut-open transition, is much the same for both partial and full agonists. This was corroborated by Mukhtasimova *et al*. [28].

## 5. The case of G-protein-coupled receptors

Given their great importance to both physiology and disease, the structure and function of G-protein-coupled receptors (GPCRs) have been at the centre of pharmacological research for decades. However, the transduction mechanisms for GPCRs are too complex to allow an experimenter to measure separately the affinity and efficacy of an agonist. The ‘cubic ternary complex’ (CTC) mechanism, which has been used to describe the interaction between agonists, GPCRs and G-proteins, contains seven free equilibrium constants in its simplest form. Six of the seven equilibrium constants reflect various aspects of efficacy; only one provides information about the agonist’s microscopic affinity and, hence, the agonist binding site. This makes structure–activity relationships for agonists of GPCRs difficult to interpret [3].

Responses to GPCR agonists that can be readily measured are often far removed from agonist binding, and several steps separate agonist binding and receptor activation from the response being measured (e.g. the accumulation of cyclic AMP). Even if one were to measure more proximal events in the transduction pathway, such as the association of a G-protein with a GPCR in response to agonist application, there are still too many steps, and too many unknowns, to make any meaningful conclusions about the microscopic affinity and efficacy of an agonist. For example, the equilibrium between the G-protein and the GPCR must be defined, and it is not even possible to define the G-protein concentration, insofar as G-proteins are membrane delimited and possibly compartmentalized, let alone account for the depletion of free G-protein as it binds to the receptor [29].

This is plain to see if one considers the maximum response elicited by an agonist in the simple CTC mechanism, that is, the maximum fraction of receptors in the agonist- and G-protein-bound active state in the presence of a saturating agonist concentration


$$p_{active}^{max}=\;\frac{E_{G}c_{G}}{1+E_{A}+c_{G}+E_{G}c_{G}},$$

where we define


$$c_{G}\equiv \frac{\left[G\right]}{K_{G}}.$$

The maximal response depends on two efficacy terms: *E*_A_, the equilibrium constant for the isomerization of the receptor between its inactive and active state with agonist alone bound; and *E*_G_, the equilibrium constant for the isomerization of the receptor between its inactive and active state when both agonist and G-protein are bound. The maximal response also depends on [*G*], the concentration of G-protein, and *K*_G_, the dissociation constant for the binding of G-protein to the agonist-bound inactive state of the receptor.

Measurements of the maximal response to an agonist in these types of experiments cannot provide the agonist’s efficacy at the level of the receptor; rather, these measurements provide information about the overall ‘coupling efficiency’ of the pathway being studied. Consequently, it is possible for an agonist that is partial at the receptor level to appear full at the cellular level, and vice versa. The agonist concentration required to elicit the half-maximal response (or half-maximal binding) is dependent on all seven free equilibrium constants in the CTC mechanism and so cannot be easily interpreted either. These experiments provide useful information about the cellular responses evoked by different agonists under different conditions but provide little information about the properties of the agonist–receptor interaction.

A great deal of our understanding of GPCR function comes from experiments that measure events downstream of receptor activation. However, an understanding of an agonist’s properties will require knowledge of the conformational changes of the receptor itself, as is the case for ligand-gated ion channels. There are currently limited methods for directly observing agonist-induced changes in the conformation of a GPCR in real time. One way to do this is to use single-molecule intramolecular resonance energy transfer measurements. Such measurements may be difficult to interpret because they depend on the relative locations of the donor and acceptor (chosen by the experimenter). How the distance between a given donor–acceptor pair relates to the various states of the receptor may not be clear as much of our understanding of agonist-induced conformational changes in GPCRs comes from static crystallographic and spectroscopic experiments. It also remains to be seen whether such experiments have the resolution required to yield estimates of the rate constants in a reaction mechanism.

Attempts have been made to fit the rate constants of realistic mechanisms of GPCR function (including multiple active receptor conformations) to experimental data [30,31], but the fits are far too over-parametrized to provide good estimates.

## 6. Conclusions

Stephenson greatly advanced pharmacology when he recognized that it is impossible to describe the action of an agonist at equilibrium with a single number; his addition of one more parameter—efficacy—provided a qualitative description of partial agonism. The simple example in §2 clearly shows that Stephenson’s framework does not allow the separate estimation of values of microscopic affinity and efficacy. These two properties of agonists are inextricably linked, and, in most sorts of experiment, they cannot be isolated from one another. Thus far, only single-channel recording of ligand-gated ion channel currents has allowed the separation of microscopic affinity and efficacy. Agonist binding experiments and structure–activity relationships are of limited use because the binding of, and response to, an agonist is influenced by *both* its microscopic affinity and efficacy.

Stephenson’s mistake remained unnoticed until 1987 [2] and is still often misunderstood even now. The original, erroneous framework still pervades pharmacology and has been propagated in lecture theatres, textbooks and the scientific literature for decades. Furthermore, more recent models of agonist action, such as those proposed by Furchgott [32] and Black & Leff [33], incorporated the same framework used by Stephenson, and so they too cannot separate microscopic affinity and efficacy. It was shown in 1987 that all of these methods provide, at best, information only on *K*_eff_, as do ligand binding experiments [2]. None can give information about the microscopic equilibrium constant, *K*_A_, which is what is needed to obtain information about the agonist binding site. Although the classical equations can, under limited conditions, take the same form as those that describe realistic mechanisms, it is agreed that equilibrium measurements cannot estimate the quantities with physical meaning—the underlying microscopic equilibrium constants [34].

Generations of students of biochemistry have been expected to understand the Michaelis–Menten mechanism, yet it is still rare for students of pharmacology to be taught its equivalent, the del Castillo−Katz mechanism. Indeed, the affinity–efficacy problem is not included in the core curricula for pharmacology undergraduates proposed by the British Pharmacological Society or by the International Union of Basic and Clinical Pharmacology (IUPHAR) [35].

We believe that the affinity–efficacy problem should be part of the core curriculum for students who study pharmacology as a science.

## Data Availability

Supplementary material is available online [36].
